# Identification of the mutual gliding locus as a factor for gut colonization in non-native bee hosts using the ARTP mutagenesis

**DOI:** 10.1186/s40168-024-01813-0

**Published:** 2024-05-23

**Authors:** Yujie Meng, Xue Zhang, Yifan Zhai, Yuan Li, Zenghua Shao, Shanshan Liu, Chong Zhang, Xin-Hui Xing, Hao Zheng

**Affiliations:** 1 Faculty of Food Science and Engineering, Kunming University of Science and Technology, Kunming, 650500 China; 2 MGI Tech, Qingdao, 266426 China; 3 Department of Entomology, College of Plant Protection, China Agricultural University, Beijing, 100083 China; 4 Institute of Plant Protection, Shandong Academy of Agricultural Sciences, Jinan, 250100 China; 5 Department of Chemical Engineering, Institute of Biochemical Engineering, Tsinghua University, Beijing, 100084 China; 6 Shenzhen International Graduate School, Tsinghua University, Shenzhen, 518055 China

**Keywords:** ARTP, Mutagenesis, Gut microbiota, MglB, Host colonization, Bumblebee, Honeybee

## Abstract

**Background:**

The gut microbiota and their hosts profoundly affect each other’s physiology and evolution. Identifying host-selected traits is crucial to understanding the processes that govern the evolving interactions between animals and symbiotic microbes. Current experimental approaches mainly focus on the model bacteria, like hypermutating *Escherichia coli* or the evolutionary changes of wild stains by host transmissions. A method called atmospheric and room temperature plasma (ARTP) may overcome the bottleneck of low spontaneous mutation rates while maintaining mild conditions for the gut bacteria.

**Results:**

We established an experimental symbiotic system with gnotobiotic bee models to unravel the molecular mechanisms promoting host colonization. By in vivo serial passage, we tracked the genetic changes of ARTP-treated *Snodgrassella* strains from *Bombus terrestris* in the non-native honeybee host. We observed that passaged isolates showing genetic changes in the mutual gliding locus have a competitive advantage in the non-native host. Specifically, alleles in the orphan *mglB*, the GTPase activating protein, promoted colonization potentially by altering the type IV pili-dependent motility of the cells. Finally, competition assays confirmed that the mutations out-competed the ancestral strain in the non-native honeybee gut but not in the native host.

**Conclusions:**

Using the ARTP mutagenesis to generate a mutation library of gut symbionts, we explored the potential genetic mechanisms for improved gut colonization in non-native hosts. Our findings demonstrate the implication of the cell mutual-gliding motility in host association and provide an experimental system for future study on host-microbe interactions.

Video Abstract

**Supplementary Information:**

The online version contains supplementary material available at 10.1186/s40168-024-01813-0.

## Background

Animals are intimately associated with highly complex and dynamic communities of microbes that inhabit virtually every surface of their bodies, especially in their digestive tracts [1]. Previous research has revealed that both the gut microbiota and the hosts they colonize profoundly influence the physiology and evolution of one another [2]. The host gut environment usually exerts relatively strong selective pressure on the gut members, driving the gut bacteria to surmount many complex challenges for successful living and replicating [3]. Therefore, the animal digestive tract houses bacterial communities whose composition is distinct from those in surrounding environments and other relative individuals in most cases [4, 5]. This suggests that host-associated bacteria maintain certain traits that enable them to colonize their hosts, which is the key evolutionary process in symbiotic relationships. Thus, identifying traits under host selection is crucial to understanding the processes governing nascent symbiotic interactions between animals and gut microbes [6]. The genetic basis of host colonization has focused on pathogenic bacteria [7], but only a few studies are available for intestinal symbionts.

Recently, experimental approaches involving the serial passage of free-living bacteria through digestive tracts have been used to explore the traits that mediate gut microbial association with hosts [8–10]. Combined with genomic sequencing of transmitted lineages, this strategy enables the identification of evolutionary changes in genotypes [11]. By experimentally evolving hypermutating *Escherichia coli* ∆*mutS* in the stinkbug *Plautis stali*, single mutations disrupting the carbon catabolite repression can make non-symbiotic strains evolve into insect mutualists [12]. Through transgenerational maintenance of ecologically distinct *Vibrio fischeri* from pineapple fish and plankton within the light organs of the squid *Euprymna scolopes*, the non-native lines exhibited mutations in the *binK* sensor kinase gene that conferred selective advantages by immune evasion [6]. Thus, single mutations of large effect can evoke broad consequences to evolve towards mutualism in bacteria.

Genome-scale random mutations are a major driving force of natural evolution; however, the low rate of spontaneous mutations is insufficient for effective screening, and large-scale or long-term host association experiments have always been required [13]. By deleting the *mutS* encoding the DNA mismatch repair enzyme, the hypermutating strains with elevated mutation rates are often used for experimental evolution studies [6, 8, 12]. Although the genetic toolsets are available for the model species, most gut bacteria cannot be genetically manipulated [14]. Atmospheric and room temperature plasma (ARTP) is a newly developed whole-cell mutagenesis tool based on glow discharge plasma [15]. The construction of mutant libraries using ARTP is used for industrial breeding, which obtains strains with higher production efficiency and tolerance to extreme environments [16, 17]. Since ARTP can cause mutagenesis while maintaining mild conditions, it provides a promising approach for exploring molecular evolutionary traits of gut microbes [14].

As members of the family Apidae, the honeybee and bumblebee species harbor similar sets of gut bacteria specific to the hosts [18]. Although they share core gut microbiota mainly composed of five phylotypes: *Gilliamella*, *Snodgrassella*, *Bifidobacterium*, *Lactobacillus* Firm4, and Firm5, phylogenetic analysis shows that strains from different bee species separate into divergent lineages [19, 20]. Previous genomic analyses show that some strains from *Gilliamella*, *Lactobacillus* Firm5, and *Bifidobacterium* exhibit different metabolic potentials for carbohydrates, which may be relevant to the adaption of bee hosts [21–23]. Interestingly, *Snodgrassella* strain wkB12 from *Bombus bimaculatus* could not colonize the *A. mellifera* gut, while cross-host microbe transfer is observed across *Apis* species [18]. Our recent study showed that *Snodgrassella* strains from different hosts exhibited a markedly different enrichment of single nucleotide polymorphisms in the type IV pili (T4P) genes, possibly associated with microbiota-host interactions [24]. Recently, protocols have been established for generating microbiota-depleted individuals of both honey and bumble bees and gnotobiotic models with defined microbial constituents [25, 26]. Thus, they provide experimentally tractable models to reveal mechanisms by which beneficial organisms colonize specific hosts.

Here, we constructed a mutant library of *Snodgrassella* strains from *Bombus terrestris* using ARTP mutagenesis. By serial passaging of replicate lines of the mutants, we identified genetic traits promoting colonization in the non-native host *Apis mellifera*. The evolved populations showed mutations in the *mglB* gene encoding the mutual gliding motility protein B, which emerged first and rapidly became fixed. Further, in vivo competition assays indicated that the point mutation may confer a competitive advantage in the non-native host, suggesting a potential role of the T4P-dependent cell motility establish of microbiota-host association.

## Materials and methods

### Isolation and whole-genome sequencing of *Snodgrassella* strain B10998

*Snodgrassella communis* strain B10998 was isolated from the gut homogenate of bumblebee, *B. terrestris*, from Jinan, China. The factory-farmed bumblebees were reared year-round in a climate-controlled room at 28 °C, 65% relative humidity, and continuous darkness. The dry canola pollen was sterilized by gamma irradiation (30 kGy), and the sterilizations were verified by plating suspensions on BHI plates and incubating them at 37 °C for 12 h. The sugar syrup (50% weight/volume, *w/v*) containing double-distilled water was filtered through 0.22-µm filters (Jet Bio-Filtration, Guangzhou, China). Bees were fed on the dry sterilized pollen and sugar syrup (50% *w/v*) ad libitum [27]. The pure culture of *S. communis* strain B10998 was isolated from bumblebee guts as previously described [26]. Glycerol stocks of gut homogenates were plated on brain heart infusion agar (BHIA) (Oxoid, Basingstoke, UK) supplemented with 5% (*v/v*) defibrinated sheep’s blood (Solarbio, Beijing, China). After 2 days of incubation at 35 °C under a CO_2_-enriched atmosphere (5%), visible colonies were identified by sequencing of the 16S rRNA gene with primers 27F (5′-AGAGTTTGATCMTGGCTCAG-3′) and 1492R (5′-GGTTACCTTGTTACGACTT-3′). Sanger sequencing was performed at Sangon Biotech (Shanghai, China). The identified isolates were then stocked in 25% (*v/v*) glycerol solution at − 80 °C. To obtain a complete and high-quality reference genome for accurate sequence alignment and mutation identification, the whole-genome sequencing was performed by the combination of short reading sequencing (Illumina platform) and long reading sequencing (Nanopore platform) at Novogene (Beijing, China) and GrandOmics (Wuhan, China). The hybrid assembly was then performed with Unicycler 0.5.0 [28], and the complete genome is deposited in NCBI database (GCF_031445945.1). To understand the relationship between strain B10998 and other *Snodgrassella* strains isolated from honeybees and bumblebees [19, 24], the phylogenetic tree was conducted with the PhyloPhlAn 3.0 under the “diversity low” parameter [29], and the iTOL web-based software was used for the visualization of the phylogenetic tree [30]. The whole-genome average nucleotide identity (ANI) was calculated using the fastANI software [31].

### Construction of the mutant library using ARTP

Mutagenesis was performed using the ARTP mutation breeding system, a newly developed whole-cell mutagenesis tool based on radio-frequency atmospheric-pressure glow discharge plasma (Additional file 3: Fig. S1). The atmospheric-pressure helium plasma jet ejected from the plasma generator mainly consists of chemical reactive species, including helium lines, oxygen atom lines, N_2_ lines, and hydroxyl (OH^−^) molecular band, irradiated on sample plates for microbial mutation [15]. The ARTP mutation breeding system has been reported to feature higher mutation rates while maintaining low treatment temperatures and employed for the mutation breeding of microorganisms [32]. We applied an ARTP setup (Luoyang Tmaxtree Biotechnology, China) to generate the input mutant library, including a plasma generator, a helium gas supply and control sub-system, and a sample plate made of stainless steel, which can be moved up and down.

To determine an appropriate exposure time, 10 µL of the bacterial suspension (~ 10^6^ CFU/mL) was spread onto the 8-mm disinfected stainless steel plates, followed by 0–20-s exposure time to the ARTP jet every 15 s. The flow rate of working helium gas was set at 10 standard L/min, and the power of radio frequency was set at 100 W. After ARTP treatment, the steel plates were shaken and eluted with sterile BHI broth, and the cells in the eluate were plated on BHIA for 48-h incubation. The numbers of control (a) and survival (b) clones were determined respectively to calculate the mortality rate by (a − b)/a × 100%. We set the treatment time to 45 s for a high mutation rate of the input mutant library. Finally, ~ 20,000 colonies were collected and mixed homogeneously as the input mutant library for the subsequent experiments.

### In vivo colonization assays in *A. mellifera*

The *A. mellifera* bees were from colonies maintained in the experimental apiary of the China Agricultural University. The microbiota-depleted bees were obtained as described by Zheng et al. [25] with modifications. Late-stage pupae were collected manually from brood frames and placed in sterile plastic bins. The pupae of *A. mellifera* emerged in an incubator at 35 °C, with a humidity of 50%. Newly emerged microbiota-depleted bees were kept in axenic cup cages, fed with sterilized sucrose syrup (50%, *w/v*) for 24 h, and divided into groups for subsequent colonization and in vivo competition assays. Before all subsequent experiments, we examined the gnotobiotic status of the bees. We picked two to three bee individuals from each cup cage and dissected the whole guts into 200 μL 1 × PBS buffer. We plated 50 μL of the gut homogenates of each bee onto the BHIA plates. We checked the plates 2–3 days after incubation, and all groups of bees showed no colony formation.

The concentrations of bacterial suspensions of the WT and the input mutant library were adjusted according to the OD_600 nm_, and colony-forming units were determined to confirm the number of cells. Subsequently, 1 mL of bacterial suspension aliquots at different concentrations (10^6^, 10^7^, 10^9^ CFU/mL) was mixed with 1 mL of sterilized sucrose solution (50%, *w/v*) and 0.3 g sterilized pollen. To assess the colonization of the strains, 15 newly emerged microbiota-depleted bees were placed into 1 cup cage, and 3 cages were set up for 2 experimental groups, WT and mutant library. For each group, bees were starved for 2–3 h and fed on bacterial suspensions for another 24 h. The intake volume of bacterial solution per bee in each cup was recorded before and after the colonization process, and the average number of ingested bacteria cells of individual bees was calculated. After colonization, the bees were given sterilized pollen and sugar water (50%, *w/v*) ad libitum for 10 days. On day 5 and day 10, 6 bees were randomly selected from each cup cage, and the entire guts were aseptically dissected by gently pulling the stingers without touching the abdomen surface using sterilized forceps [23]. Subsequently, the dissected guts were crushed in 100 μL 25% (*v/v*) glycerol using an electric tissue grinder (OSE-Y30; Tiangen Biotech, Beijing, China). A dilution of bacterial samples was plated on BHIA plates supplemented with 5% (*v/v*) defibrinated sheep’s blood, and the colonization levels of bees were determined by counting colony-forming units.

Since an increased colonization level was observed, we further confirmed the colonization levels when bees were fed a higher number of mutant cells (average 10^9^ per bee) in 5 replicating cup cages. Around 30 newly emerged microbiota-depleted individuals were placed into 1 cup cage as a lineage, and each bee was fed on 10^9^ bacteria cells. On day 5 and day 10 after colonization, 12 bees from each lineage were randomly selected. Their whole intestines were combined into a 2-mL tube containing 1.2 mL 1 × PBS, then homogenized using the electric tissue grinder to recover the bacterial populations. We obtained 10 samples from all 5 replicating cup cages on the 2 time points (D5, D10).

### Serial in vivo passage of the mutant library

**Fig. 1:**
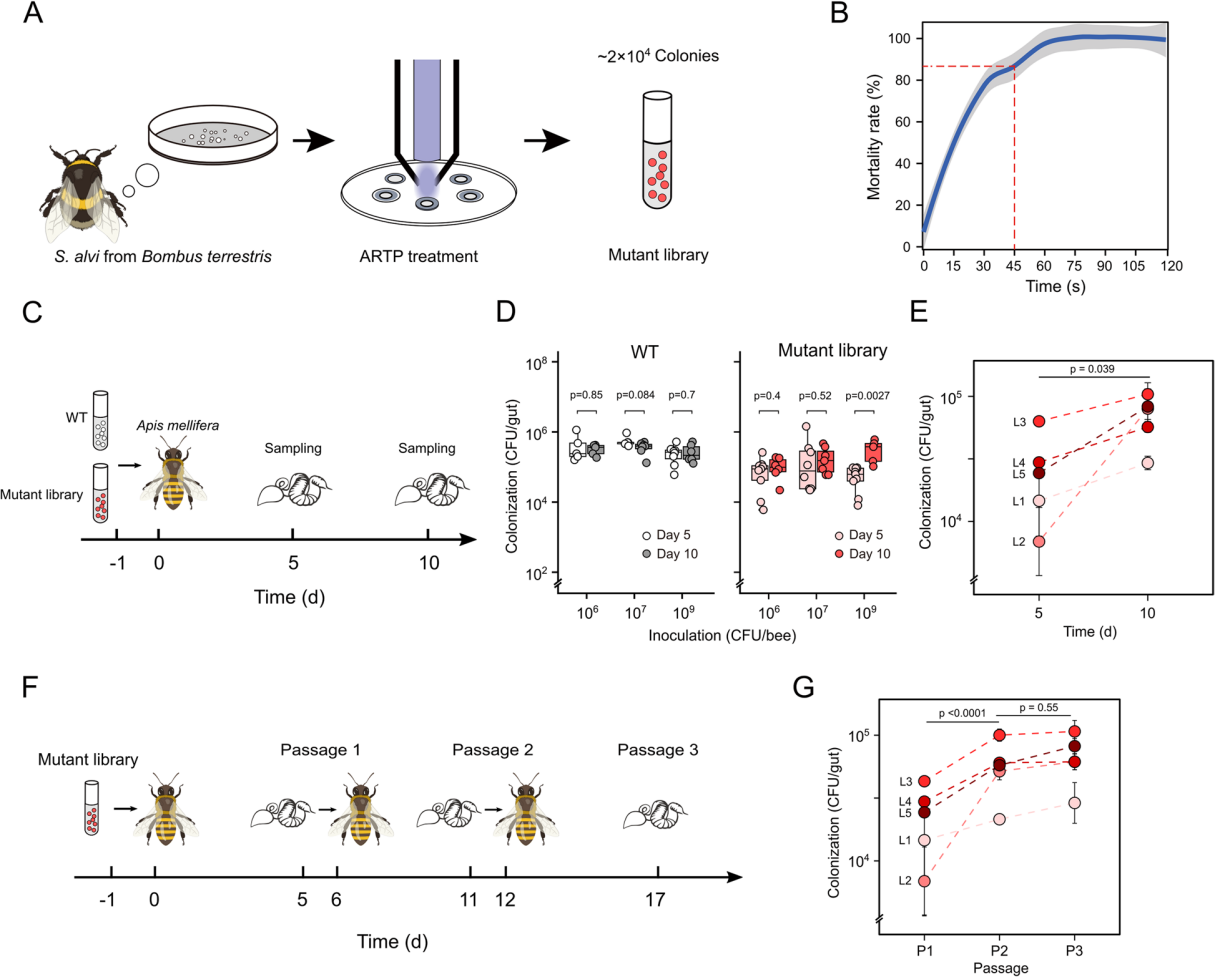
Colonization of *Snodgrassella communis* mutants obtained from ARTP treatment in non-native host species. **A** Schematic diagram of the input mutant library generation using the ARTP biological breeding system. **B** Effect of ARTP treatment on the mortality rate of *S. communis*. **C** Schematic of in vivo colonization assays. Age-controlled bees were inoculated with different amounts of WT bacteria and input mutant library (10^6^, 10^7^, 10^9^ CFU). **D** Changes in the bacterial loads of bee individuals (*n* = 6) with different inoculation levels. Statistical analysis was performed using Student’s t-test. **E** Evaluation of the bacterial colonization for bees fed with 10^9^ CFU bacterial mutants. Each dot represents a bee lineage. Statistical analysis was performed using two-way ANOVA. **F** Schematic of the serial passage experiment. The bees were first colonized with the input mutant library, and the gut homogenates were used for subsequent serial transfer. **G** Evaluation of the bacterial colonization for bees during the passage experiment. Each dot represents a bee lineage. Statistical analysis was performed using two-way ANOVA

To further identify traits that promote host association of the mutants, we performed serial passaging experiments in *A. mellifera* in 5 lineages (Fig. 1F). For each generation, we serially passaged the mutation library through microbiota-depleted bee hosts, each time specifically using gut-colonized populations as inoculum for the subsequent passage. For every cup cage, 15 newly emerged microbiota-depleted bees were placed into 1 cup cage, and the individuals were firstly inoculated by bacterial suspensions from the input mutant library mixed with 1 mL sterilized sucrose solution and 0.3 g sterilized pollen for 24 h. The intake volume of bacterial solution per bee in each cup was recorded before and after the colonization process. After colonization, the bees were given sterilized pollen and sugar water (50%, *w/v*) ad libitum for 5 days, and then the intestines of bees were removed by dissection. While *Snodgrassella* is mainly localized to the ileum region, it can also be found in the midgut and the rectum [33]. Therefore, the whole intestines from all bees in the same cage were dissected and mixed into a 2-mL tube containing 1.2 mL 1 × PBS; then, they were homogenized using the electric tissue grinder as P1 samples. The number of *S. communis* populations in different samples was determined by plating a small aliquot (20 μL) of the mixed gut sample on BHI plates. Then, the colonies were counted and collected for further metagenomic sequencing. Half of each homogenate (600 μL) was mixed with 600 μL of sterile 50% glycerol and was stored at − 80 °C. The remaining homogenates were centrifuged (5000 × *g*, 5 min), resuspended, and added to the food of the next cages of microbiota-depleted bees as the inoculum. Similarly, after 1-day colonization and 5-days feeding with sterilized pollen and sugar water (50% *w*/*v*) ad libitum, the whole guts of bees in the same cage were dissected, pooled together, and homogenized as P2-group samples for subsequent plate counting and bacterial colonies collection. Half of each homogenate was stored at − 80 °C, and the remaining was used as the inoculum for the next cup of bees. We also repeated the colonization process to obtain P3-group samples.

### Metagenomic sequencing and analysis

We obtained 15 samples from 5 lineages of the serial passage experiment and 5 samples (P1, P2, P3) from the gut samples after 10-day colonization (D10 group). The bacterial populations were recovered by plate cultivation, and ~ 300 single colonies from each plate were collected by scrapping and resuspending in 1 × PBS for DNA extraction. The Ezup Column Bacteria Genomic DNA Purification Kit (Sangon Biotech, Shanghai, China) was used for DNA extraction for pooled colonies and the input mutant library. NEBNext UltraTM II DNA Library Prep Kit for Illumina (New England Biolabs, MA, USA) was used for the generation of sequencing libraries, and Qubit 3.0 Fluorometer (Life Technologies, Grand Island, NY, USA) and Agilent 4200 (Agilent, Santa Clara, CA, USA) system were used for library quality assessment. The libraries were then sequenced on the Illumina HiSeq platform with 150-bp paired-end reads. We obtained about 1 Gbases sequencing data for each sample, with an average sequencing depth of 500 × for *S. communis* B10998. Fastp [34] was used for adaptor trimming and quality control of the raw sequencing data.

To confirm whether the recovered *Snodgrassella* strains derive from our inoculum, we profiled the species-level community of the populations using the Metagenomic Intra-Species Diversity Analysis System (MIDAS) pipeline [35] with the custom database that included genomes of pure isolates from the guts of both bumblebees and honeybees [24]. The relative abundance of bacteria species was estimated by mapping quality-filtered reads to the database of phylogenetic marker genes using HS-BLASTN, and the results were combined using the “merge_midas.py species” module. Mutations in all sequenced populations were identified using the *breseq* 0.37.0 pipeline [36], and our de novo assembled high-quality genome of strain B10998 was used as the reference. The pipeline uses a reference sequence-based mapping strategy, which includes evaluating new sequence junctions supported by split-read alignments and tracking multiple mapped reads to predict point mutations and structural mutations from short-read DNA resequencing data. Mutations were called only for regions covered by a minimum of 20 reads. The polymorphism prediction may be prone to false positives because of many biases in NGS data. Thus, low-frequency base substitutions (< 5%) based on read alignment evidence are treated as false positives [36].

To initially compare the mutational diversity between the groups, we calculated the number of polymorphic mutations and the frequency-weighted average, *F* = ∑*f*_*i*_, with *f*_*i*_ for all the detected mutations of each sample. As described by Turner et al. [37], we also used the Bray–Curtis dissimilarity index, considering the frequency of the mutations in each population to measure the genetic similarities between populations. For genes that mutated more than once in the same population, we used the sum of the different mutations for each gene in each population. To further assess the mutation pattern in different populations, the SNPEff software [38] was used for functional annotation of coding single nucleotide polymorphisms (SNPs). We first used the gdtools utility GF2VCF to convert the *breseq* variant call files from gd format into vcf files (the required input for SNPEff). In addition, the distribution of variant types in different samples was visualized by using the R-tidyverse package.

### Allele identification by sanger sequencing

The mutations in the *mglB* gene predicted by *breseq* were confirmed by Sanger sequencing of the amplicon sequencing of a 250-bp fragment that contains the mutation sites. The L2-group and WT bacterial populations were recovered by plate cultivation, and eight bacteria colonies from each plate were identified by colony PCR. A pair of primers targeting the 235th nucleotide position within the functional domain, B01065-F (5′-CAGCTCTTTCAGGGCTCGTT-3′) and B01065-R (5′-TGCATTACAAAGCGGCATGG-3′), were designed. We performed the sequence alignment using ClustalW2 [39] and recorded the genotypes of different colonies.

### Prediction of mutation affection on the protein stability

The SMART (Simple Modular Architecture Research Tool) web server was used to annotate the protein domains of MglB [40]. According to the result of BLASTp, the protein MXAN_5770 (PDB 7CY1, resolution 2.19Å) was used as a template to homology model the structure of the *mglB* in *S. communis* B10998 with 99% confidence using Phyre2 (version 2.0) in intensive mode [41]. Based on the predicted PDB file, the impact of the variant on *mglB* structure was analyzed using Missense3D [11], which predicts structural changes leading to the disruption of alpha helices or beta sheets, alterations in inter-residue interactions in the structures, and unfavorable energy changes that affect the protein’s function. The variant site locations were submitted to the Protein Variation Effect Analyzer (PROVEAN) [42], which introduces a versatile alignment-based score to predict the damaging effects of single amino acid substitutions. We also carried out a free energy (ΔΔ*G*) calculation for point mutations in the available protein structure using MAESTRO [43] to evaluate the change in protein stability upon the mutation.

### Phylogenetic analysis of MglB

The representative arrangements of the mgl operons from bacteria were identified based on the dataset from Wuichet et al. [44]. We performed Blastp using MglB from *S. communis* B10998 as a query against genomes of all *Snodgrassella* strains from our custom bee gut bacteria database [24]. We also obtained homologous sequences from the UniRef 90, including hits with *e* values < 0.0001. The multiple-sequence alignments were built using the L-INS-i algorithm of the MAFFT (version 7.487) [45]. Phylogenetic trees of MglB were built with FastTree (version 2.1.3) [46] using the slow option and were visualized using the R package ggtree [47].

### Colony expansion and competition assays in vivo

To test whether the missense mutations identified in the *mglB* of *S. communis* alter the bacterial phenotype, we recovered bacterial populations from the samples. The freezer stocks were streaked onto plates and incubated for 2 days, and 24 isolated colonies were picked for purification. We exacted the DNA of 9 pure isolations and submitted them to the Illumina platform for sequencing. After adaptor trimming and quality control, mutations in all sequenced isolations were identified using the *breseq* 0.37.0 pipeline [36] with the B10998 genome as the reference. We successfully screened 2 strains, including mutant SA01065, which harbored the designated variant in *mglB* (G78R) and a background mutant SA01065’ harboring all variants except for the mutation on *mglB* (Additional file 3: Fig. S2).

Motility assays were performed as described by Keilberg et al. [48] with modification. Bacterial cells of *S. communis* (SA01065, SA01065’, WT) were harvested and resuspended in BHI broth to a final density of ~ 10^9^ cells/ml. Subsequently, 5-µL aliquots of cells were placed on 0.5% and 1.5% BHIA and incubated at 35 °C. After 72 h, the colony edges were observed using a microscope (SMZ745T, Nikon).

To confirm that the mutation in *mglB* influenced the gut colonization characteristics of *S. communis*, we assayed by competing the mutant SA01065 against the background strain SA01065’ and the WT in both honey (*A. mellifera*) and bumble (*B. terrestris*) bee hosts. The strains (SA01065, SA01065’, WT) were grown on BHIA plates supplemented with 5% (*v/v*) defibrinated sheep’s blood. The colonies were scraped and resuspended in 1 × PBS. The optical density (OD_600 nm_) was adjusted according to the subsequent conditions.

For competition in *A. mellifera*, the SA01065 mutant was adjusted to an OD_600 nm_ of 0.005 as the reference. The WT and the background SA01065’ were adjusted to an OD_600 nm_ of either 0.005, 0.05, or 0.5 as the competitors. Competing strains were mixed at 1:1 (competitor: reference). Then, 1 mL of the mixture was added to 1 mL of sterile sugar water (50%, *w/v*) and 0.3 g of sterile pollen and fed to newly emerged *A. mellifera* for 24 h. Conversely, for competition in *B. terrestris*, the WT and the background strain SA01065’ were adjusted to an OD_600 nm_ of 0.005 as the reference. The SA01065 mutant was adjusted to an OD_600 nm_ of either 0.005, 0.05, or 0.5 as the competitor. To obtain microbiota-depleted *B. terrestris*, we picked pupae from dark cocoons in the bumble bee colonies, as previously reported [49]. Each cocoon was opened with sterilized forceps, and mature pupae were aseptically removed from their cocoons with sterilized forceps. The pupa was then placed into sterile plastic bins and maintained at 28 °C, 65% relative humidity, to obtain microbiota-depleted individuals. Single bumblebees were kept separately in every cup to ensure the generation of microbiota-depleted individuals.

After colonization, all bee individuals were given sterilized pollen and sugar water ad libitum. Six bees per experiment were dissected on day 5 after colonization. The relative abundance of the two competing strains was analyzed by Sanger sequencing, which can help identify specific mutation loci of *mglB* in genomes. Eight individual colonies from each sample were determined by colony PCR reactions with the designed primers mglB-F/R (Additional file 3: Fig. S3). We assigned a classification to each bacterial colony based on the type of base at the mutant position we identified for estimating the proportion of different strains in the population.

Furthermore, the amplicon sequencing targeted to the mutation site was used to detect the community composition. The gut homogenates for six honeybees or bumblebees per group were mixed, and the DNA was extracted using the FastPure Blood/Cell/Tissue/Bacteria DNA Isolation Mini Kit (Vazyme Biotech., Nanjing, China). The DNA amplification was performed with designed primer amp-F (5′-TTGGCCTCATGAGTAGTGGT-3′), amp-R (5′-ACGGTTACCCAAAGACAGCA-3′), and 2 × Phanta Flash Master Mix (Dye Plus) (Vazyme Biotech., Nanjing, China). After purification with the FastPure Gel DNA Extraction Mini Kit (Vazyme Biotech., Nanjing, China), the amplification products were used for the library preparation with MGIEasy Universal DNA Library Prep Set (MGI Tech., Shenzhen, China). The libraries were then sequenced on the DNBSEQ-E25 platform (2 × 150 bp) (MGI Tech., Shenzhen, China). After quality control with fastp [34], the obtained reads were then analyzed with the custom script “04-Amplicon.sh”, which was available on GitHub (https://github.com/mengyujiee/ARTP).

## Results

### Specific ARTP-induced mutations potentially contributing to the colonization of *Snodgrassella* in non-native hosts

The strain *Snodgrassella* B10998 was isolated from the gut homogenate of *B. terrestris*, and the results of phylogenetic analysis and ANI comparison showed that this strain belongs to *Snodgrassella communis*, which is formally described and named recently [19] (Additional file 3: Fig. S4). We first constructed an input mutant library of wild-type (WT) *Snodgrassella communis* B10998 isolated from *B. terrestris* using the ARTP mutation breeding system (Fig. 1A). Cell suspensions with ~ 10^4^ CFUs were placed on the steel plates. They were exposed to the ARTP jet (radio frequency power = 100 W). First, to determine an appropriate exposure time for ARTP, we evaluated the mortality rate of *S. communis* exposed to a gradient of irradiation time (Fig. 1B). The mortality rate of the strain increased with the treatment time prolonged and reached approximately 85% when the bacterial cells were submitted for 30 s of irradiation. Almost 99% of the cells died as the exposure time exceeded 60 s. Thus, we set the treatment time to be 45 s with a cell lethality percentage of ~ 90% to provide a high mutation rate [50]. The treated cells were then incubated on BHIA for 48 h. Finally, ~ 20,000 single colonies were collected and mixed homogeneously as the input mutant library for the subsequent experiments.

To explore the genetic mechanisms contributing to colonization, we assessed the colonization potential of the input mutant library in the non-native host, *A. mellifera*. Different amounts of cells (10^6^, 10^7^, 10^9^ CFU) of WT and the mutants were inoculated into microbiota-depleted *A. mellifer*a individuals, and the gut microbial loads were quantified at day 5 and day 10 by plate counting (Fig. 1C). When *A. mellifera* were fed WT strains from bumblebee with different inoculation size, the bacterial load was maintained at 10^5^–10^6^ CFU/gut, which is much lower than the colonization levels of the native strains (10^7^–10^8^ CFU/gut) [18, 51]. There were no significant differences between day 5 and day 10 for all inoculation groups (Student’s *t*-test, *p* = 0.85, *p* = 0.084, *p* = 0.7) (Fig. 1D), indicating that the colonization level had reached a maximum after 5 days [51]. For bees fed the input mutant library, the overall colonization levels were lower or equal to the WT strain, indicating that most of the random mutations by ARTP are neutral or deleterious [52]. However, we noticed that the bacterial loads increased over time (Student’s *t*-test, *p* = 0.0027) when bees were fed a high number of mutant cells (10^9^ CFU/bee) (Fig. 1D). This suggests a genetic factor potentially contributing to a competitive advantage in gut colonization. Thus, we specifically evaluated the bacterial colonization for bees fed a high amount of the input mutant library (10^9^ CFU/bee) in 5 replicating lineages, and each lineage represents a cup cage of bees (10–15 individuals) fed the same input library. The bacterial densities were calculated based on plate counting for the pooled gut samples from the same cages. The same increasing trend was observed over time (two-way ANOVA, *p* = 0.0039) (Fig. 1E).

To further identify traits that promote gut colonization of the mutants, we serially passaged the input mutant library (10^9^ CFU) in five replicating lineages (Fig. 1F). We serially passage the mutation libraries through microbiota-depleted bee hosts, each time specifically using gut-colonized populations as inoculum for the subsequent passage. Similarly, we observed significant increases (two-way ANOVA, *p* < 0.0001) in colonization density during the first passage, but it remained unchanged (two-way ANOVA, *p* = 0.55) for the next round of passage (Fig. 1G). Together, these results suggest the emergence of mutations potentially contributing to the competitive advantage of *S. communis* in the non-native host species.

### Mutations underlying *Snodgrassella* gut colonization

To investigate the genetic determinants of gut colonization, we recovered the colonized population from all 5 replicate lineages of different groups. First, we sequenced the metagenomes of ~ 300 pooled colonies from each lineage and classified the species composition using the MIDAS pipeline (Additional file 1: Dataset S1). It showed that almost all recovered colonies belong to *Snodgrassella communis*, a recently described species specific to *Bombus* species [19]. Only small fractions of the reads are assigned to other bee gut bacteria, including *Lactobacillus kunkeei* (0.05–0.22%), *Gilliamella apis* (0.07%), *Gilliamella apicola* (0.02%), and *Spiroplasma melliferum* (0.04%). The metagenomic reads were then aligned to the genome of *S. communis* B10998 wild type for further mutation identification using *breseq*. We detected 25 mutations in the input mutant library (Additional file 2: Dataset S2), and the frequency of most loci was between 5 and 10%. These mutations are located in the coding regions of genes associated with stress response (*qseC*, *fpr*) and DNA damage repair (*lrp*, *recC*), suggesting that ARTP induces DNA damage and potentially leads to recombinant DNA repair processes [53–56]. Notably, we noticed that two mutations within *rpsG* and *glcA* occurred with a 100% frequency, which may contribute to DNA damage repair and recombination and oxidative resistance, respectively [57, 58]. These two mutations were not present in the wild-type strain but were fixed in the input mutant library and introduced into the serial passage process.

**Fig. 2:**
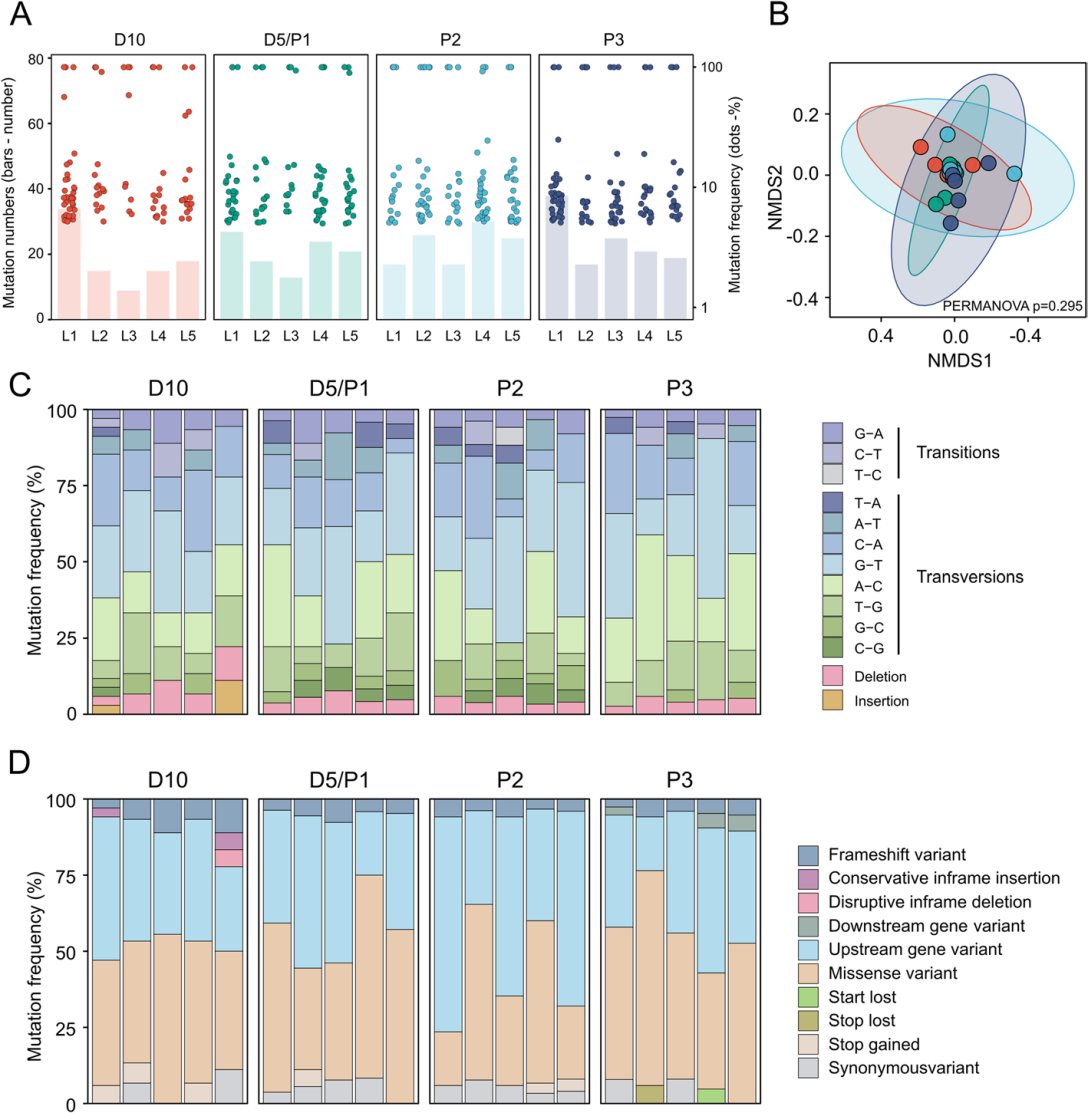
Characterization of the mutations during in vivo passage. **A** The total number of mutations (y-axis, bars) and frequencies of mutations (z-axis, dots) for all samples; each sample is a mixture of ~ 300 bacterial colonies from the gut homogenate of 10–15 bee individuals. **B** The non-metric multidimensional scaling analysis (NMDS) using the Bray–Curtis dissimilarity index considering the frequency of the mutations in each sample. The differences were analyzed for statistical significance using permutational multivariate ANOVA (PERMANOVA). **C**, **D** Overview of mutation genotype (**C**) and the predicted effect of the mutations (**D**). Each bar presents situations of the mutations in every lineage

We then quantified the number and frequency of mutations in the different samples from the serial passage (Fig. 2A). No differences in the mutation numbers (one-way ANOVA, *p* = 0.62) and average mutation frequencies (one-way ANOVA, *p* = 0.457) were observed across samples collected on day 5, day 10 and from different passages (Additional file 3: Fig. S5). To further assess the mutation pattern in different populations, we annotated the types and functions of variants using SnpEff [38] (Additional file 2: Dataset S2). Although different groups did not split (PERMANOVA, *p* = 0.295) based on the genetic similarity calculated by the Bray–Curtis index (Fig. 2B), subtle differences in the coding effect of mutation genotypes were identified. SNPs are the most prevalent, and insertion mutation was only found in the D10 group (Fig. 2C). Although below 10% of mutations are likely to cause loss of functions (start lost, stop lost, stop gained, or frameshift mutations), most of the mutations identified are missense, which might result in changes of gene expression or protein activity (Fig. 2D). In summary, these results indicate that the frequency and pattern of mutations do not alter significantly during the in vivo passaging.

### Mutation in *mglB* confers competitive advantages in the non-native host

**Fig. 3:**
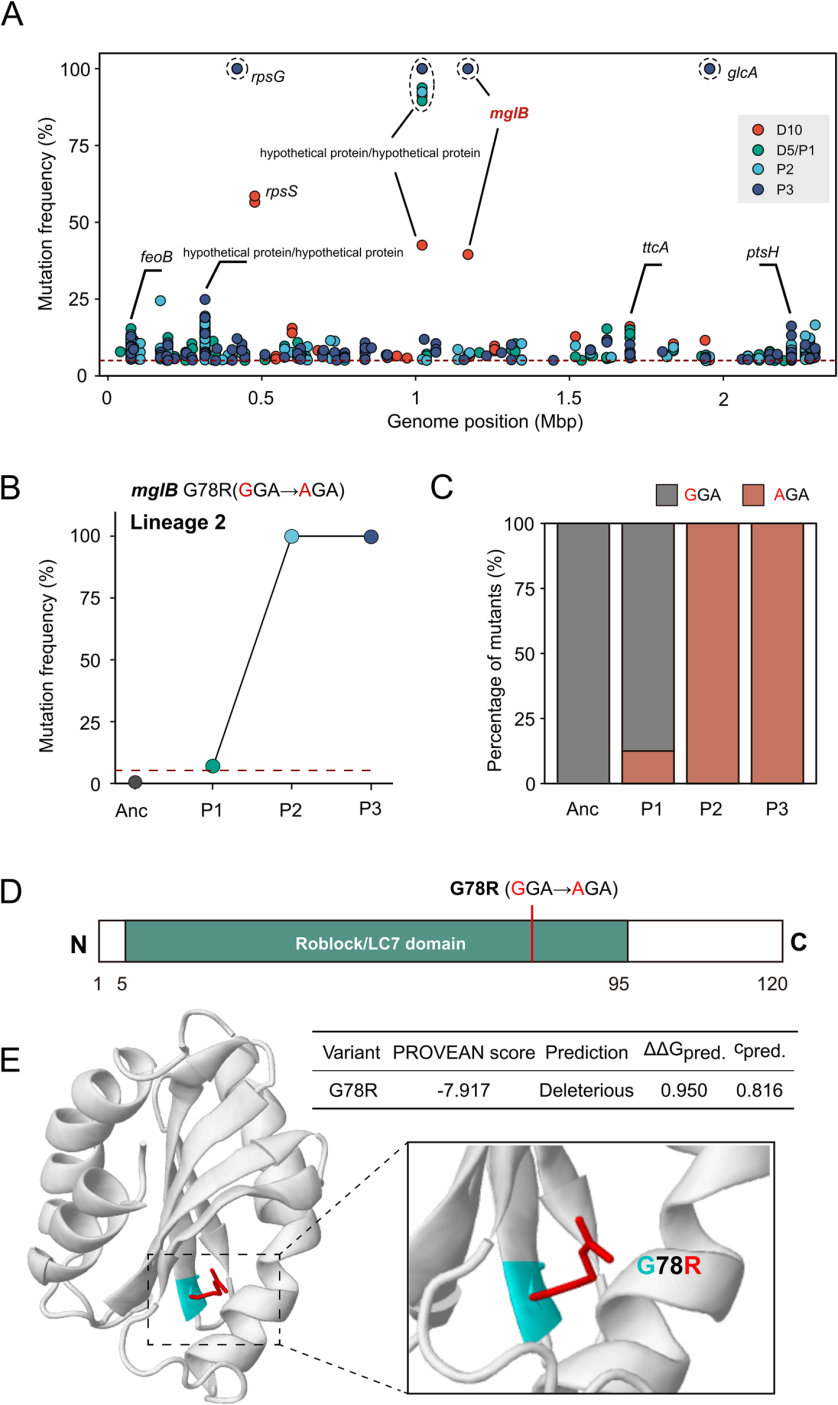
The point mutation occurring in *mglB* may confer competitive advantages. **A** The positions and frequencies of the detected mutations. Each data point represents a mutation, and colors indicate different groups. Dashed circles indicate overlapping data points. **B** Mutation frequency of *mglB* G78R(GGA → AGA) in lineage 2 samples. **C** Verification of the presence and percentage of the variants by PCR from isolated strains targeting the polymorphism in *mglB*. **D** Protein architecture of MglB from *S. communis* B10998. Specific mutations in the evolved isolates are labeled. **E** The Missense3D analysis of the variant potentially causing structural alteration. The structure of MglB was predicted by modeling against the solved PDB crystal structure (PDB number 7CY1, resolution 2.19 Å)

To further identify the potential hotspots of variants along the genome for host colonization, we analyzed the positions and frequencies of the mutations that arose in our experimental process (Fig. 3A). The frequencies of most identified mutations ranged from 5 to 20%. These mutations are widely scattered across the whole genome of the populations from different groups, and no bias regarding genome position was identified. We noticed that the mutations within *rpsG* and *glcA*, already detected in the input mutant libraries, were retained in the following passages with 100% frequency (Fig. 3A, Additional file 2: Dataset S2). Additionally, several sites in *feoB*, *rpsS*, *ttcA*, and *ptsH* were hit more frequently and reached high frequency, but these mutations are not shared across passages (Additional file 2: Dataset S2). Notably, a base insertion in *mglB* encoding the mutual gliding motility protein B occurred (39.5%) in lineage 5 on day 10 (Additional file 2: Dataset S2). Moreover, the frequency of a point mutation in *mglB* was only 6.9% in passage 1 of lineage 2 (P1L2) but rose rapidly to 100% in passage 2 and was maintained in passage 3 (Fig. 3B). This suggests that the *mglB* mutation may confer a competitive advantage in gut colonization. The presence and percentage of the variants were directly verified by PCR from single isolated strains using primers targeting this polymorphism (Additional file 3: Fig. S3). By sequencing eight clones from each group, we found that the mutation frequency was highly consistent with the results predicted by sequence alignment (Fig. 3C; Additional file 3: Fig. S3).

Annotation of the *mglB* gene predicted it to be a 121-amino acid protein containing the functional domain—roadblock/LC7 (Robl_LC7), a widespread superfamily involved in regulating NTPases and the modulation of dynein functions [59]. The identified mutation occurred at the 235th nucleotide position within the functional Robl_LC7 domain, resulting in one non-synonymous amino acid substitution (Fig. 3D). We predicted the structure of MglB by modeling against the solved PDB crystal structure (PDB number 7CY1, resolution 2.19 Å) (Fig. 3E). The Missense3D analysis of the missense variant showed that this substitution replaces a buried uncharged residue with a charged residue, which may cause structural alteration.

Moreover, the mutation happened at a position in the transition of two anti-parallel β-sheet sandwiched and is likely deleterious with a PROVEAN score of − 7.917 [42]. According to the MAESTRO prediction, it may also strongly impact protein stability (ΔΔG = 1.738, confidence = 0.873) [43]. Therefore, the mutation identified in *mglB* is likely to alter the protein markedly, probably linked to the host colonization.

### The mgl operon in *Snodgrassella* strains

**Fig. 4:**
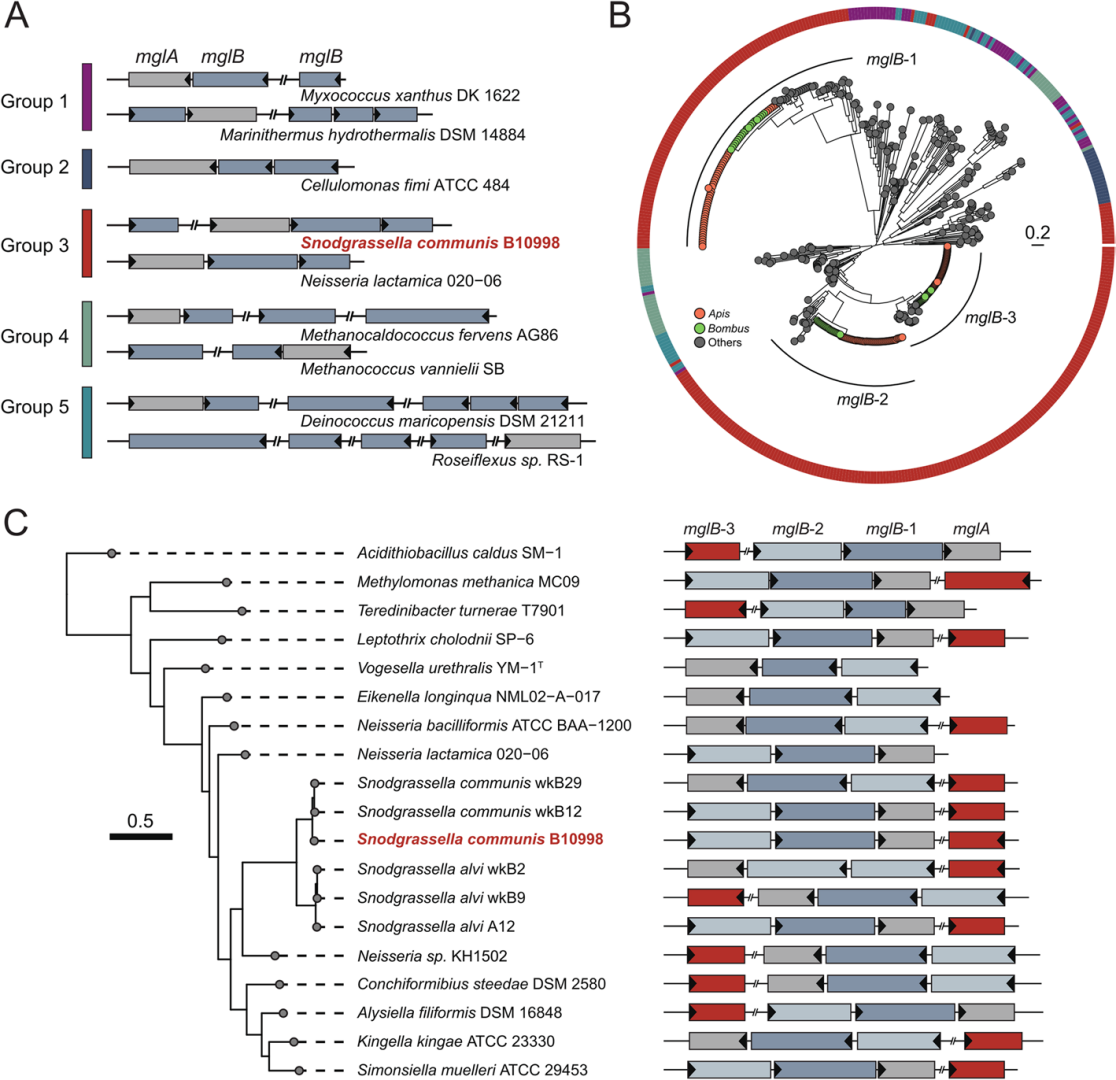
The mgl operon in *Snodgrassella*. **A** Distribution patterns of the mgl operon in different bacterial groups. Bacteria were grouped according to the phylogeny of the MglA sequences [44]. **B** Maximum-likelihood phylogeny of the MglB from *Snodgrassella* strains (see also Additional file 3: Fig. S4). **C** Genome-wide phylogenetic tree of a subset of bacterial strains containing the group 3 mgl and the gene arrangement of the loci

MglB is a major component of the mgl operon, which is essential to regulating motility, cell polarity, predation, development, and antibiotic resistance of bacteria [44]. The operon contains co-transcribed open reading frames of the mutual gliding motility A (MglA) and its GTPase-activating protein MglB. The mgl operon of *S. communis* B10998 contained only one *mglA* and three *mglB* genes in the genome. Genes encoding MglA and MglB homologs are often coupled in the genomes [59]; however, one of the *mglB* genes from B10998 (*mglB*-3) was located ~ 10^6^ bp away from the locus and was deemed orphan. Notably, the identified mutation related to the host colonization was in the coding region of the orphan *mglB*-3. The uncoupled MglA and MglB sequences are also observed in a subset of systems with multiple *mglB* genes (Fig. 4A) [44].

The MglA family members have been classified into five distinct groups [44]. The *mglA* from *S. communis* B10998 belongs to group 3, which is confined to specific taxonomic groups, including *Neisseria*, *Stenotrophomonas*, and *Xanthomonas*. Furthermore, the mgl operon was identified in all genomes of *S. communis* strains isolated from *A. mellifera* and *Bombus* spp. hosts. All bee gut *S. communis* contained three *mglB* genes, and they formed three distinct clades in the phylogenetic tree together with sequences derived from *Neisseria* (Fig. 4B; Additional file 3: Fig. S6). Interestingly, the orphan *mglB* was more closely related to the *mglB*-2 that is adjacent to the *mglA*, while the other coupled *mglB*-1 was distantly related. We further identified the arrangements of the mgl operons from group 3 (Fig. 4C). Almost all of them harbored three *mglB* genes, and the orphan *mglB* genes were phylogenetically closely related, suggesting an ancient duplication or horizontal transfer of an *mglB* gene [44]. Moreover, we performed sequence alignment for MglB-3 of *Snodgrassella* from honeybees and bumblebees (Additional file 3: Fig. S7), and the identified mutation in MglB-3 (G78R) is not found in the isolate genomes.

### The mutation identified in *mglB* provides a fitness advantage

Compared to other paralogs, the orphan *mglB* represents an important paradigm of gene duplication and divergence [60]. Previous studies document that it plays an important role in regulating the type IV pili-dependent cellular motility, and the *ΔmglB* cells exhibit altered colony morphology and gliding motility [44]. Therefore, we tested whether the missense mutation we identified in the *mglB* of *S. communis* can alter the phenotype. We generated arrayed libraries by picking single colonies from different passaged populations and obtained the whole-genome sequences of different colonies. Cryo-archived passaged population samples from D5/P1, P2, P3, and D10 were plated for isolation, and the genomes of nine colony-purified isolates were re-sequenced. One mutant, SA01065, which harbored the designated variant in *mglB* (G78R), was selected from the L2 sample, and a background mutant, SA01065’, harboring all variants except for the mutation on *mglB* was used as a control (Additional file 3: Fig. S2).

**Fig. 5:**
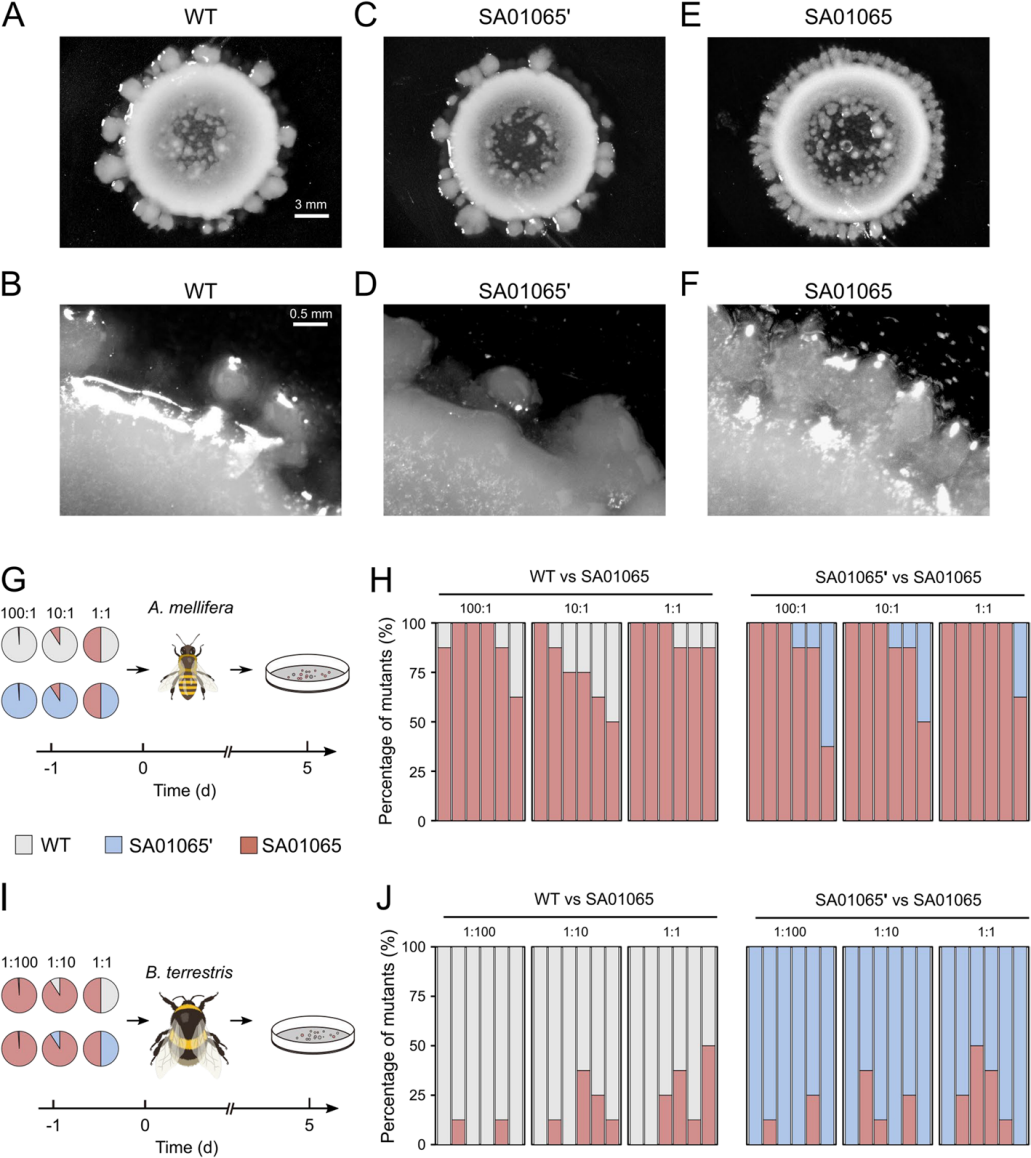
The mutation identified in *mglB* affects cell motility and in vivo competitive advantage. **A**–**F** Colony expansion assay of the WT (**A**, **B**), the background mutant SA01065’ (**C**, **D**), and mutant SA01065 (**E**, **F**) on 0.5% agar. G–J In vivo competition assays in the non-native *Apis mellifera* (**G**, **H**) and the native host *Bombus terrestris* (**I**, **J**). Two sets of experiments were conducted, including WT versus SA01065 and SA01065’ versus SA01065. Single-colony PCR was performed to identify the different variant types of *S. communis* B10998 in the population

We performed colony expansion assays by incubating isolates on agar plates. Although on 1.5% agar medium, the WT and the two mutants did not show different colony morphology (Additional file 3: Fig. S8), the SA01065 colony exhibited a completely different morphology from the other two strains on the 0.5% agar (Fig. 5A–F). The SA01065 mutant formed denser dendrite-like structures spreading across the surface of the media (Fig. 5E, F), implying an alteration in cell motility.

Finally, to confirm that the mutation in *mglB* influenced the gut colonization characteristics of B10998, we assayed for competitive advantage by competing the mutant SA01065 against the background strain SA01065’ and the WT in both honey and bumble bee hosts. First, we co-inoculated the SA01065 with the background strain SA01065’ or the WT in the non-native host, *A. mellifera*. We kept the number of bacteria in the inoculum constant for SA01065 but provided SA01065’ or WT at different ratios (1:1, 10:1, 100:1) relative to SA01065 to test if SA01065 can colonize despite a numerical disadvantage (Fig. 5G). After the 5-day colonization, we quantified the colonization level by plate counting. There was no significant difference in the absolute bacterial density between the groups from the same bee species (one-way ANOVA; *p* = 0.164 in *A. mellifera*; *p* = 0.795 in *B. terrestris*) (Additional file 3: Fig. S9). The genotypes were determined by amplifying with the designed primers and Sanger sequencing. For each gut sample, we sequenced 8 randomly picked colonies, and we totally analyzed 48 colonies from 6 biological replicates for each treatment (Fig. 5G, H). Despite their low proportion in the initial intake population, almost all recovered colony-forming units from both setups were of the SA01065 mutant (Fig. 5H). This indicates that the mutant SA01065 can overcome a large numerical disadvantage and completely out-compete the background strain and the WT in *A. mellifera*. However, when we retested in the native bumblebee host, SA01065’ and WT became dominant in any single gut, even when SA01065 was more abundant at a ratio of 100:1 in the inoculum (Fig. 5I, J). In addition, we also confirmed the community composition with amplicon sequencing (Additional file 3: Fig. S10). We amplified a 144-bp region covering the suspected mutation in *mglB*. It showed that SA01065 dominated the gut of *A. mellifera* but was out-competed by the background strain and WT in *B. terrestris* (Additional file 3: Fig. S10), which agrees with the results of Sanger sequencing. Taken together, the results of the reciprocal colonization confirmed that the mutations in *mglB* provide a competitive advantage for the non-native bacteria strain in *A. mellifera*.

## Discussion

In this study, we constructed a model system for studying the host association traits of gut symbionts. We found a point mutation in *mglB* that dominated the population during passaging in the non-native bee host. Further experiments suggest that the mutation can alter the colony morphology and provide a competitive advantage in the *A. mellifera* gut. We provide experimental evidence that the mutual gliding motility protein MglB may contribute to colonization in non-native hosts by altering the motility phenotype.

We subjected the ARTP-treated input mutant library of *S. communis* to the non-native host, *A. mellifera*, to track the potential molecular factors related to host association. Most corbiculate bees, including honeybees and bumblebees, retain similar core bacterial lineages. However, different bee species possess host-specific bacterial species and strains [61]. Although cross-host microbe transfer is observed across *Apis* species, host fidelity is rather strict in transfers between *Apis* and *Bombus* species [18, 20, 51]. We found that neither the wild type (~ 10^6^ CFU/gut) nor the mutant library (~ 10^5^ CFU/gut) of the non-native strain reached the colonization level of the native strain (~ 10^8^ CFU/gut) (Fig. 1D) [18]. The lack of generalist strains capable of colonizing the distantly related honeybee and bumblebee hosts could be attributed to the significant differences in the physiologies that are too substantial to overcome [18].

Additionally, we noticed that the colonization level of the input mutant library is lower than the wild type (Fig. 1D). ARTP mutagenesis primarily operates through the damage to bacterial genetic material by highly active particles, leading to diverse random mutations via the SOS repair mechanism [32]. Here, the in vitro mutagenesis is performed without selective pressures. We suspect most mutations would be neutral or deleterious, and the advantageous mutations are rare [52]. Notably, the bacterial loads increased over time when the honeybees were fed a high number of mutant cells. Effectual alleles must confer a selective advantage in the symbiotic association and arise early during population growth to survive extinction under the host-imposed bottlenecks [6]. Clear population bottleneck must occur during our in vivo passage as only ~ 10^5^ CFU per bee out of the input library (10^9^) can colonize. The mutation in *mglB* rose from a low frequency (6.9%) to 100% within two generations. However, this mutation was not detected in the input mutant library directly after the mutagenesis. Since ARTP may create DNA damage and lead to the introduction of mutations during the consequent DNA repair [15], the mutation observed in *mglB* might emerge during the following in vivo passages. Alternatively, this could be attributed to the sensitivity limits of the *breseq* pipeline [36], or it might be that the *mglB* is a compensatory mutation that arises later in the mutated strains, offering them an advantage in colonizing the gut [8].

Mutual gliding motility (mgl) is a locus essential for bacterial motilities, which mainly consists of two components, *mglA* and *mglB*, encoding the small Ras-like GTPase mutual gliding motility A (MglA) and its GTPase activating protein mutual gliding motility B (MglB) [62]. The locus is present in ancient, deep-branching lineages of Bacteria and Archaea, especially widely detected in pathogens such as *Neisseria*, *Stenotrophomonas*, and *Xanthomonas* [44]. Mgl is a component of the polarity control system, which acts as an intracellular switch to control bacterial motility and thereby regulates cooperative movement in the bacterial community [63]. It profoundly affects biofilm formation by influencing the transition from individual to collective movement patterns [64]. Consistently, experimental evidence shows that deletion of the *mglA* and *mglB* genes individually or in tandem reduces bacterial adhesion and biofilm formation [65]. *Snodgrassella* normally attaches to the inner intestinal wall to form biofilm in the bee ileum [61], suggesting that the abilities of adhesion to host epithelial cells and biofilm formation are essential for their in vivo colonization. A genome-wide Tn-seq analysis showed that lack of biofilm formation was detrimental in vivo and associated with diminished host colonization of *Snodgrassella* in *A. mellifera* [66]. Thus, we hypothesize that the mgl locus is also involved in the colonization of *Snodgrassella*, potentially affecting the capabilities of adhesion and biofilm formation. Interestingly, we revisited the raw dataset of the TnSeq libraries and found that *mglB*-1 (SALWKB2_RS10975), *mglB*-2 (SALWKB2_RS10980), and *mglB*-3 (SALWKB2_RS04330) of *Snodgrassella* are all beneficial for gut colonization [66]. In addition, the *mglB* genes of *Snodgrassella* strains from different bee hosts were clustered separately in the phylogenetic tree, suggesting that the mgl locus may affect the unique ecological niche and host colonization of *Snodgrassella*.

There is usually only one coding region of *mglA* and multiple *mglB* coding regions in the bacterial genome [44]. Some *mglB* genes were co-transcribed with *mglA*, while some exist independently and are defined as “orphan” *mglB*. In *Snodgrassella*, all strains from the bee gut microbial database showed identical gene arrangements, which include two *mglB* genes co-transcribed with *mglA* and one orphan *mglB*. We identified that the host-associated mutation occurred in the orphan *mglB*. Unlike the coupled one, the orphan MglB lost its GAP activity against MglA, while it interacts with the coupled MglB homodimers to regulate cell polarity and motility. The Δ*mglB* mutant of *M. xanthus* showed reduced T4P-dependent and gliding motility in the colony expansion assays [60]. In our study, we did not observe different colony morphology of the mutants on the 1.5% agar, but the motile “flares” of cells were observed at the mutant colony edge on the soft 0.5% agar, indicating that the mutation in the orphan *mglB* alters the type IV pili (T4P)-dependent motility of *Snodgrassella* [67]. T4P are common bacterial surface appendages and encode membrane-associated proteins capable of binding bacteria to host cells, which affects the colonization of some bacteria through surface adhesion and motility regulation [66]. The genome-wide Tn-seq analysis has documented that the T4P genes of *Snodgrassella* are beneficial for gut colonization [66]. In addition, our previous study showed that *Snodgrassella* strains exhibit significantly different SNPs enrichment in the T4P genes, and the distribution of SNPs deviates markedly between genetically different hosts, suggesting that T4P is involved in microbiota-host interactions of *Snodgrassella* [24]. The activated MglA-GTP can stimulate the T4P formation and function, and MglB ensures T4P unipolarity by excluding MglA-GTP from the lagging pole [48]. Thus, the mgl operon may affect the *Snodgrassella* colonization by regulating T4P-dependent motility and biofilm formation.

In *A. mellifera*, high levels of strain-level diversity exist, even within single-host individuals. These closely related strains with a gANI > 95% may adopt specific spatial and metabolic niches [68]. Although only one *Snodgrassella* species exists in *A. mellifera*, five *Snodgrassella* species have been identified from *Bombus* hosts [19]. Our metagenomic analysis showed that all the recovered *Snodgrassella* populations belong to the *Bombus*-specific *S. communis*, suggesting that they are not likely contaminated by the endogenous strains from honeybees during the serial passages. However, *Snodgrassella* always co-colonizes the bee ileum with *Gilliamella*, which supports each other’s persistence [51, 68]. In this study, we passaged the *Snodgrassella* without the participation of *Gilliamella*, so we could not illustrate how the evolutionary pressures of *Gilliamella* may have impacted the colonization of *Snodgrassella* in the bee gut. *Snodgrassella* form contiguous biofilms with *Gilliamella* along the length of the ileum [68]. Without *Gilliamella*, the capability to establish biofilm on its own may be a crucial factor for the successful colonization of *Snodgrassella*. Moreover, *Snodgrassella* and *Gilliamella* form a syntrophic network for partitioning metabolic resources. For example, *Gilliamella* may acquire amino acids and pyrimidines from *Snodgrassella*, and they contribute vitamins to each other [51]. Therefore, a divergent genetic basis conferring selective advantages and host colonization in *Snodgrassell*a may be identified when passaging with the existence of *Gilliamella* strains.

## Conclusions

In this study, we adopted the ARTP technique to obtain mutant libraries containing many mutant cells to explore the potential alleles associated with host colonization. By colonizing and passaging the *S. communis* strains within the non-native host *A. mellifera*, one experimental line rapidly dominated by a specific single mutation in *mglB*, and mutants showed a definite competitive advantage over the wild type in the competition experiments. The alleles potentially affected the bacterial T4P system recruiting in the correct pole in motility, suggesting the possible involvement of the cell motility against the host immunity system in establishing successful host colonization. Overall, our results demonstrate the adaptability of innovative ARTP technology in accelerating the host-associated studies of gut symbiosis. The ARTP, as a powerful mutagenesis method, may contribute to the progress of a comprehensive study of an organism mutation and evolution.

### Supplementary Information

**Additional file 1:** **Dataset S1.** Strain-level composition of samples from different groups.

**Additional file 2:** **Dataset S2.** Polymorphic variant calling on the *Snodgrassella* populations during the serial colonization in vivo of the mutant library.

**Additional file 3:** **Fig. S1**. (A-B) Pictures of the ARTP mutation breeding system, the front view (A), and the operation chamber (B). The equipment used to generate the helium radio-frequency ARTP jet consists of a 13.56-MHz power supply, a co-axial-type plasma generator, a helium gas supply and control subsystem, and a stainless-steel sample plate that can be moved smoothly in the vertical direction to adjust the stand-off distance between the plasma torch nozzle exit and the sample plate. (C-D) The procedure of mutagenic treatment consisted of several steps. Firstly, an aliquot of pretreating liquid was taken and coated uniformly on the surface of the slide and placed in the chamber of the ARTP mutagenesis operation instrument. Then, the distance between the slide and the plasma emitter jet orifice was adjusted, the constant power output power was 120 W, and the gas flow rate was 10 SLM. The processing time of mutation was chosen and adjusted. Bacterial cells are exposed to plasma for random mutagenesis. **Fig. S2**. Mutations in the three strains used in colony expansion experiments and in vivo competition experiments. **Fig. S3**. Design of primers (A) and results of sequence alignment (B) for allele identification by Sanger sequencing. **Fig. S4**. (A) Whole-genome phylogenetic tree based on representative isolates’ genomes of *Snodgrassella*. The tree was rooted with the sequence of *Simonsiella muelleri* ATCC 29453. The phylogenetic clusters are noted based on the results of Cornet et al. (2022). (B) Heatmaps show the values of pairwise ANI of representative isolates’ genomes of *Snodgrassella*. **Fig. S5**. Mutation numbers (A) and the average mutation frequencies (B) in different groups. Each data point indicates an independent lineage. Statistical analysis was performed using oneway ANOVA. **Fig. S6**. Maximum-likelihood phylogeny of the MglB from *Snodgrassella* strains. **Fig. S7**. Visual representation of MglB-3 sequences from (A) *Apis mellifera* and (B) *Bombus* species, prepared using WebLogo (https://weblogo.berkeley.edu/logo.cgi). **Fig. S8**. Colony expansion assay of the WT (A), the background mutant SA01065’ (B), and mutant SA01065 (C) on 1.5% agar. **Fig. S9**. The absolute bacterial density of *Apis mellifera* (A) and *Bombus terrestris* (B) for the in vivo competition assays. **Fig. S10**. In vivo competition assays in the non-native *Apis mellifera* (A) and the native host *Bombus terrestris* (B). Two sets of experiments were conducted, including WT versus SA01065 and SA01065’ versus SA01065. The amplicon sequencing was performed to identify the different variant types of *Snodgrassella communis* B10998 in the population.

## Data Availability

Sequencing data of the metagenome and amplicons have been deposited under BioProject PRJNA1014770. All scripts and code used in this article have been uploaded to GitHub (https://github.com/mengyujiee/ARTP).

## Abbreviations

ARTP: Atmospheric and room temperature plasma
T4P: Type IV pili
BHIA: Brain heart infusion agar
SNP: Single nucleotide polymorphism
MIDAS: Metagenomic Intra-Species Diversity Analysis System
