# Supplementary material for: Identification of the mutual gliding locus as a factor for gut colonization in non-native bee hosts using the ARTP mutagenesis
Source: Microbiome. 2024 May 23;12:93. doi: 10.1186/s40168-024-01813-0 (PMC11112851; doi:10.1186/s40168-024-01813-0)
Supplement: Supplementary file 4 — Additional file 3: Fig. S1. (A-B) Pictures of the ARTP mutation breeding system, the front view (A), and the operation chamber (B). The equipment used to generate the helium radio-frequency ARTP jet consists of a 13.56-MHz power supply, a co-axial-type plasma generator, a helium gas supply and control subsystem, and a stainless-steel sample plate that can be moved smoothly in the vertical direction to adjust the stand-off distance between the plasma torch nozzle exit and the sample plate. (C-D) The procedure of mutagenic treatment consisted of several steps. Firstly, an aliquot of pretreating liquid was taken and coated uniformly on the surface of the slide and placed in the chamber of the ARTP mutagenesis operation instrument. Then, the distance between the slide and the plasma emitter jet orifice was adjusted, the constant power output power was 120 W, and the gas flow rate was 10 SLM. The processing time of mutation was chosen and adjusted. Bacterial cells are exposed to plasma for random mutagenesis. Fig. S2. Mutations in the three strains used in colony expansion experiments and in vivo competition experiments. Fig. S3. Design of primers (A) and results of sequence alignment (B) for allele identification by Sanger sequencing. Fig. S4. (A) Whole-genome phylogenetic tree based on representative isolates’ genomes of Snodgrassella. The tree was rooted with the sequence of Simonsiella muelleri ATCC 29453. The phylogenetic clusters are noted based on the results of Cornet et al. (2022). (B) Heatmaps show the values of pairwise ANI of representative isolates’ genomes of Snodgrassella. Fig. S5. Mutation numbers (A) and the average mutation frequencies (B) in different groups. Each data point indicates an independent lineage. Statistical analysis was performed using oneway ANOVA. Fig. S6. Maximum-likelihood phylogeny of the MglB from Snodgrassella strains. Fig. S7. Visual representation of MglB-3 sequences from (A) Apis mellifera and (B) Bombus species, prepared using W [file 40168_2024_1813_MOESM3_ESM.pdf]

Supplementary Information

Identification of the mutual gliding locus as a factor for gut colonization in non-native bee hosts using the ARTP mutagenesis

Yujie Meng[1,2], Xue Zhang[3], Yifan Zhai[4], Yuan Li[2], Zenghua Shao[2], Shanshan Liu[2], Chong Zhang[5], Xin-Hui Xing[6], Hao Zheng[1,*]

[1]Faculty of Food Science and Engineering, Kunming University of Science and Technology, Kunming 650500, China

[2]MGI Tech, Qingdao 266426, China

[3]Department of Entomology, College of Plant Protection, China Agricultural University, Beijing 100083, China

[4]Institute of Plant Protection, Shandong Academy of Agricultural Sciences, Jinan 250100, China

[5]Department of Chemical Engineering, Institute of Biochemical Engineering, Tsinghua University, Beijing 100084, China

[6]Shenzhen International Graduate School, Tsinghua University, Shenzhen 518055, China

*Corresponding author:

Hao Zheng, zhenghao05@gmail.com

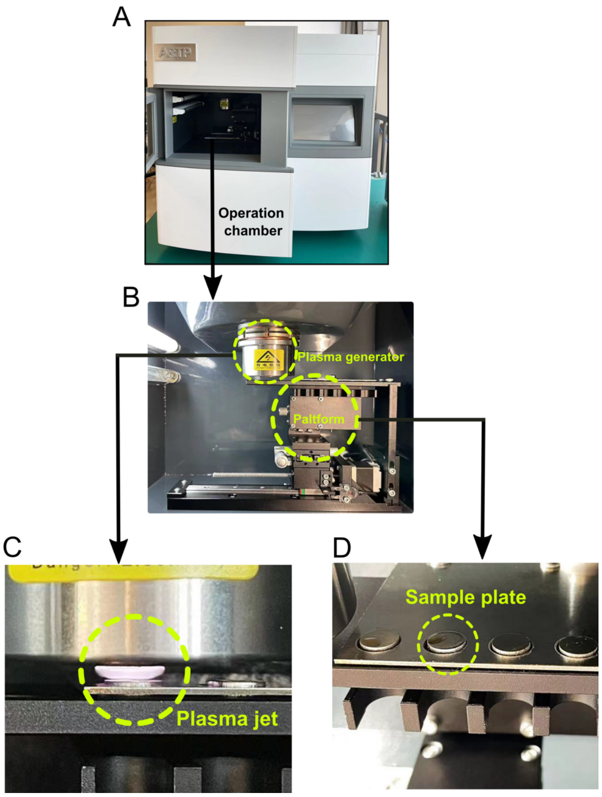

Figure S1. (A-B) Pictures of the ARTP mutation breeding system, the front view **(A),** and the operation chamber **(B)**. The equipment used to generate the helium radio-frequency ARTP jet consists of a 13.56-MHz power supply, a co-axial-type plasma generator, a helium gas supply and control subsystem, and a stainless-steel sample plate that can be moved smoothly in the vertical direction to adjust the stand-off distance between the plasma torch nozzle exit and the sample plate. **(C-D)** The procedure of mutagenic treatment consisted of several steps. Firstly, an aliquot of pretreating liquid was taken and coated uniformly on the surface of the slide and placed in the chamber of the ARTP mutagenesis operation instrument. Then, the distance between the slide and the plasma emitter jet orifice was adjusted, the constant power output power was 120 W, and the gas flow rate was 10 SLM. The processing time of mutation was chosen and adjusted. Bacterial cells are exposed to plasma for random mutagenesis.

Gene	Mutation	WT	SA01065'	SA01065
rpsG	G81V(GGT→CTT)		√	√
00946/00947	intergenic (-195/+225)		√	√
mglB	G78R(GGA→AGA)			√
glcA	Δ1 bp (1009/1644 nt)		√	√

Figure S2. Mutations in the three strains used in colony expansion experiments and *in vivo* competition experiments.

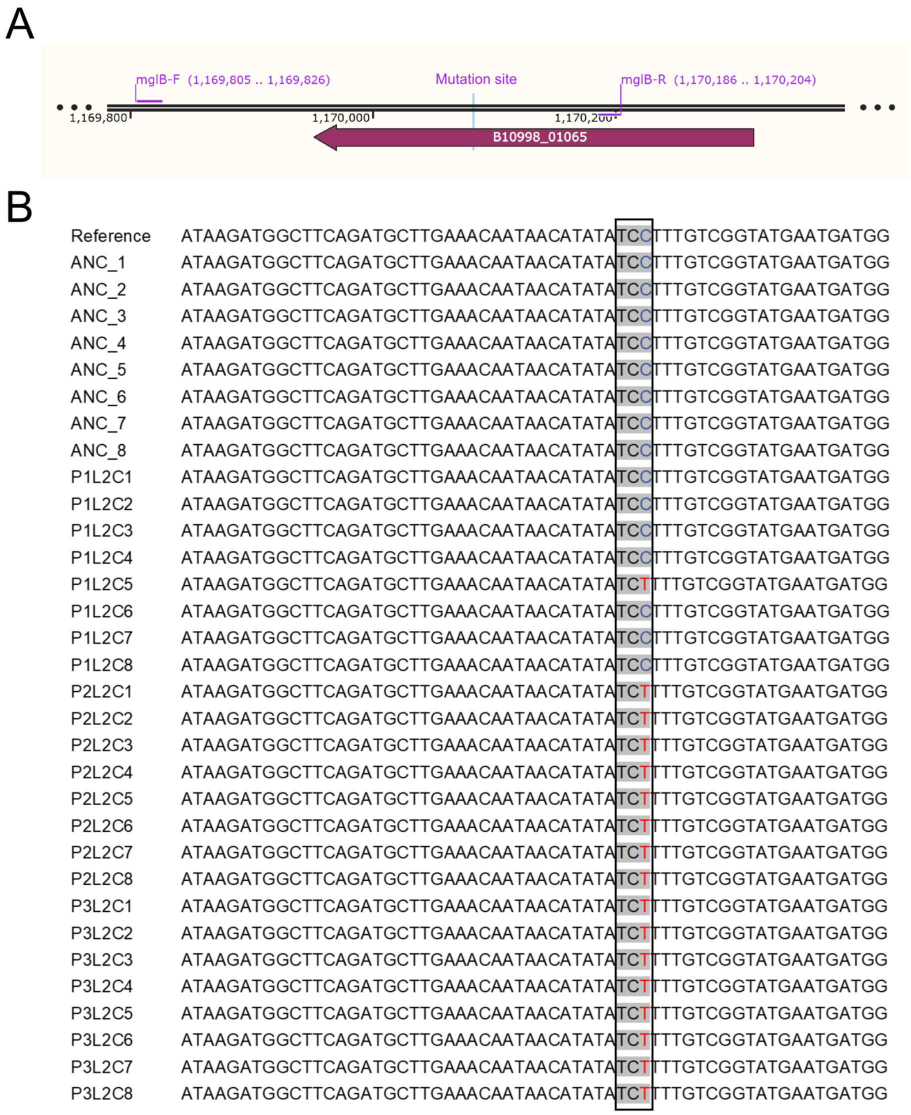

Figure S3. Design of primers **(A)** and results of sequence alignment **(B)** for allele identification by Sanger sequencing.

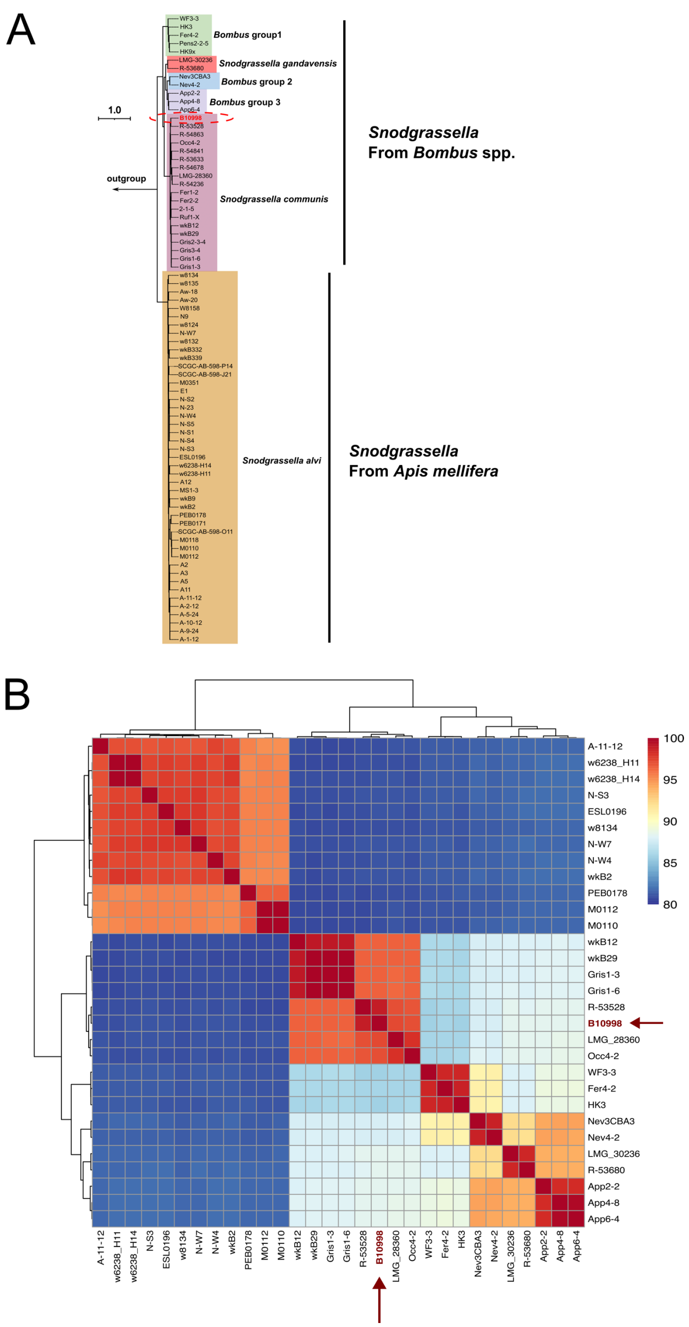

Figure S4. (A) Whole-genome phylogenetic tree based on representative isolates' genomes of *Snodgrassella*. The tree was rooted with the sequence of *Simonsiella muelleri* ATCC 29453. The phylogenetic clusters are noted based on the results of Cornet et al. (2022).

(**B**) Heatmaps show the values of pairwise ANI of representative isolates' genomes of *Snodgrassella.*

Cornet L, Cleenwerck I, Praet J, Leonard RR, Vereecken NJ, Michez D, et al. Phylogenomic analyses of *Snodgrassella* isolates from honeybees and bumblebees reveal taxonomic and functional diversity. mSystems. 2022;7:e01500-21.

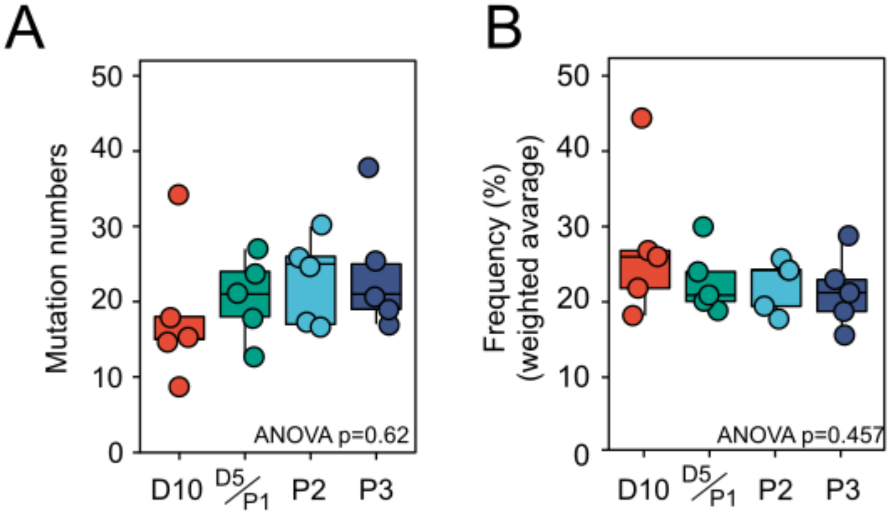

Figure S5. Mutation numbers **(A)** and the average mutation frequencies **(B)** in different groups. Each data point indicates an independent lineage. Statistical analysis was performed using one-way ANOVA.

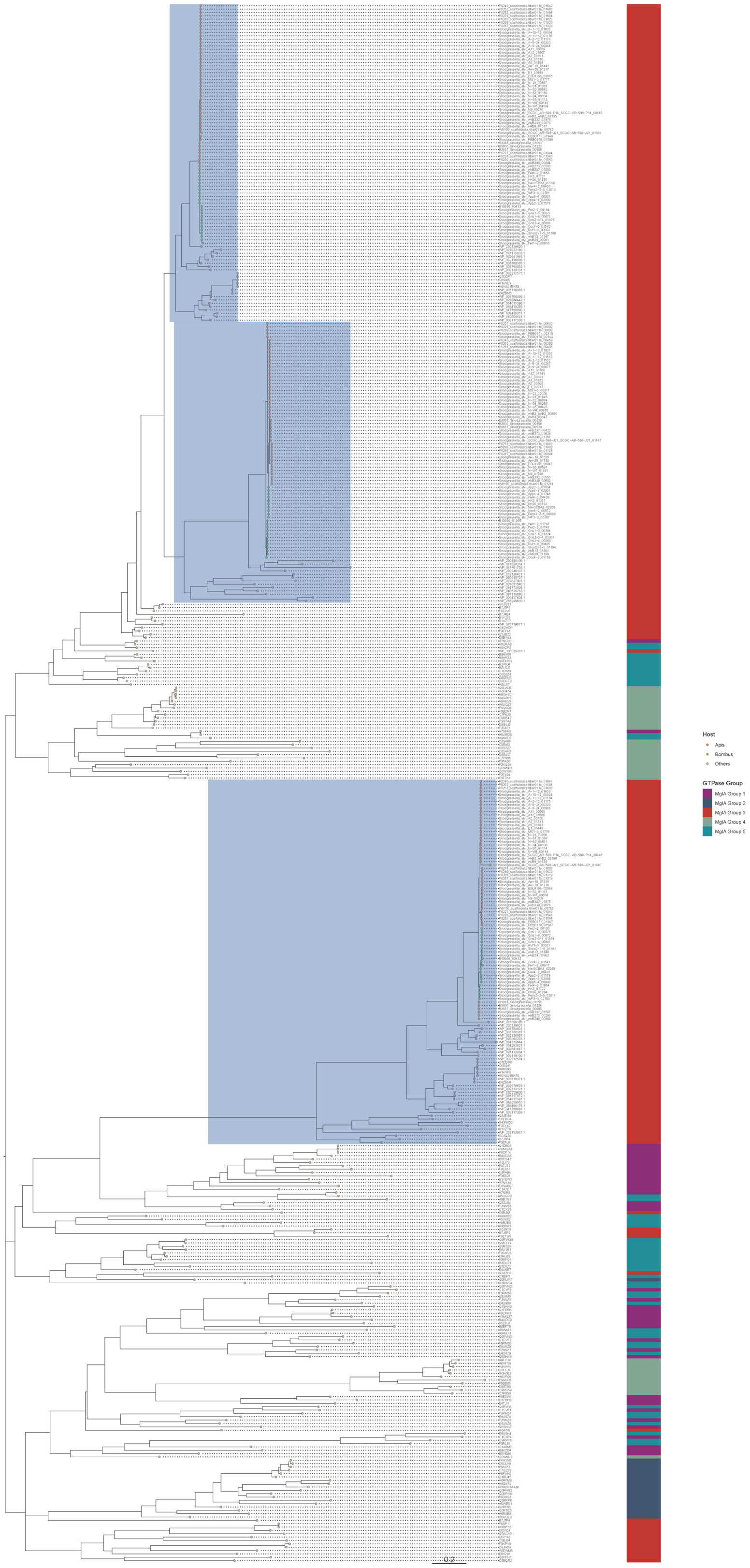

Figure S6. Maximum-likelihood phylogeny of the MglB from *Snodgrassella* strains.

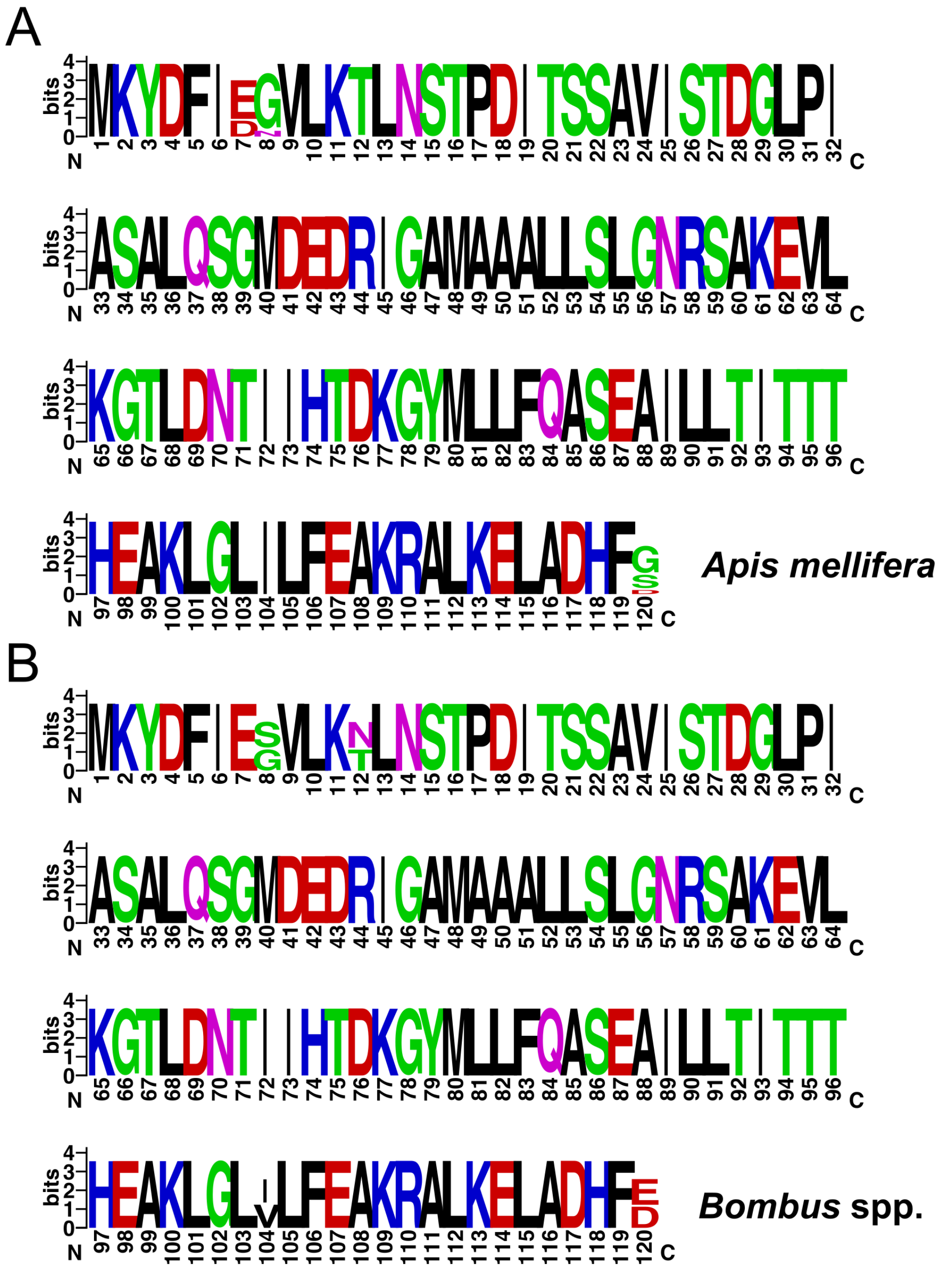

Figure S7. Visual representation of MglB-3 sequences from **(A)** *Apis mellifera* and **(B)** *Bombus* species, prepared using WebLogo (https://weblogo.berkeley.edu/logo.cgi).

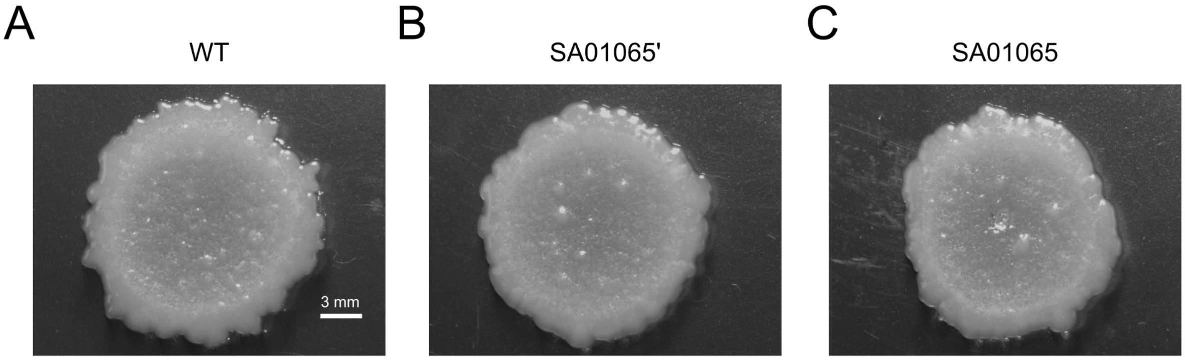

Figure S8. Colony expansion assay of the WT **(A)**, the background mutant SA01065’ **(B)**, and mutant SA01065 **(C)** on 1.5% agar.

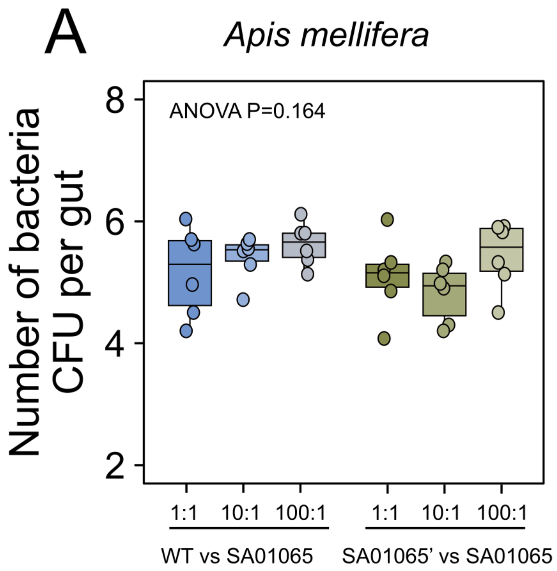

Figure S9. The absolute bacterial density of *Apis mellifera* **(A)** and *Bombus terrestris* **(B)** for the *in vivo* competition assays.

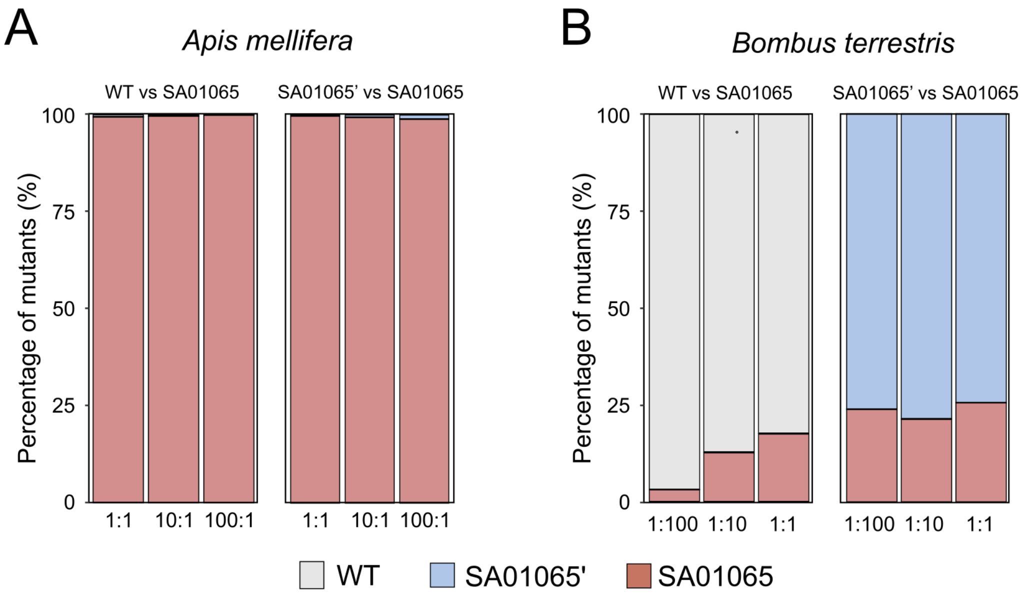

Figure S10. *In vivo* competition assays in the non-native *Apis mellifera* **(A)** and the native host *Bombus terrestris* **(B)**. Two sets of experiments were conducted, including WT versus SA01065 and SA01065' versus SA01065. The amplicon sequencing was performed to identify the different variant types of *Snodgrassella communis* B10998 in the population.

Additional files:

Additional file 1: Dataset S1. Strain-level composition of samples from different groups.

Additional file 2: Dataset S2. Polymorphic variant calling on the *Snodgrassella* populations during the serial colonization *in vivo* of the mutant library.
